# Health Experts’ voice for Healthier Choice – a call for Zero Air Pollution in the Western Balkans

**DOI:** 10.1093/eurpub/ckac129.145

**Published:** 2022-10-25

**Authors:** M Jevtic, V Matkovic

**Affiliations:** 1 Faculty of Medicine University of Novi Sad, Novi Sad, Serbia; 2 Institute of Public Health of Vojvodina, Novi Sad, Serbia; 3 Health & Environment Alliance HEAL, Brussels, Belgium

## Abstract

In 2019, the European Environment Agency has shown that the fine particulate matter PM2.5 caused more than 25 000 premature deaths in six Western Balkans countries alone, namely Albania 4 000, Bosnia and Herzegovina 5 900, Kosovo* 2 800, Montenegro 900, North Macedonia 3 400 and Serbia 11 400. In the same year, more than 2 200 lives were lost due to nitrogen dioxide (NO2) and ozone (O3) pollution. Air pollution and climate change are major health problems in the region. Health experts in the Western Balkans region have joined forces to highlight the importance of air quality actions and achieving zero air pollution as a prevention intervention for public health. We brought together a call for the Western Balkans policy-makers to invite them to commit to full alignment of all national air quality standards with the World Health Organization guidelines, to establish regional intersectoral cooperation to accelerate the moving to zero pollution, to including health authorities, public health institutes, and medical societies, patient representative and all health care experts and providers; to end direct or indirect public subsidies of polluting processes, especially fossil fuel activities such as coal power plants; to finalise the process of the ratification of the Convention on Long-range Transboundary Air Pollution and its protocols; to support modelling to establish economy-wide emission reduction commitments for the five main pollutants; to develop and implement Air Quality Strategies; to increase the uptake of Best Available Technologies (BAT) in accordance with the Industrial Emissions Directive; to establish an adequate air quality monitoring system, and to including through accreditation of air quality monitoring networks. Public health experts from the WB, united in the regional call, highlight the urgent need for improvements in air quality in the region together with a zero air pollution objective and a timeline to reach it.

